# Epicardial fat thickness is associated to type 2 diabetes mellitus in Korean men: a cross-sectional study

**DOI:** 10.1186/s12933-015-0210-7

**Published:** 2015-05-03

**Authors:** Hyejin Chun, Eunkyung Suh, A Ri Byun, Hae Ran Park, Kyung Won Shim

**Affiliations:** Health Promotion Center, Ewha Womans University Mokdong hospital, Ewha Womans University School of Medicine, Anyangcheon-ro, Yangcheon-gu, Seoul, 158-710 South Korea; Department of Family Medicine, Chaum Hospital, CHA University, 4-1, Cheongdam-dong, Gangnam-gu, Seoul, 135-948 South Korea; Departments of Family Medicine, Ewha Womans University Mokdong hospital, Ewha Womans University School of Medicine, 1071, Anyangcheon-ro, Yangcheon-gu, Seoul, 158-710 South Korea

**Keywords:** Epicardial fat tissue, Diabetes, Low-dose chest CT, Visceral fat

## Abstract

**Background:**

Visceral fat, including epicardial fat (EF) is recognized as a responsible factor of cardiovascular disease. The aim of this study was to investigate the relationship between EF and diabetes in Korean men.

**Methods:**

EF thickness was measured in the left main coronary artery fat tissue (LMCA-fat) by low-dose chest CT scans in 1,048 Korean men (age above 20 years). LMCA-fat values were divided into quartiles and the prevalence of diabetes was analyzed based on the quartiles of LMCA-fat values using logistic regression.

**Results:**

There were significant correlations between LMCA-fat and body mass index (r = 0.169, p = 0.004), waist circumference (r = 0.172, p < 0.001), fasting glucose (r = 0.106, p = 0.037) and HbA1c (r = 0.176, p < 0.001). The patients in the higher LMCA-fat quartiles were associated with higher prevalence of diabetes (p for trend <0.001). Even after adjustment for multiple covariates, this association still remained statistically significant (p for trend = 0.022). The highest LMCA-fat quartile group was significantly associated with diabetes compared to the lowest quartile group. (OR = 3.26, 95% CI = 1.17-9.12).

**Conclusion:**

These findings indicate that increased EF thickness is independently associated with the prevalence of diabetes in Korean men.

## Background

Obesity and insulin resistance are important risk factors of diabetes and atherosclerosis [1]. In particular visceral fat is known to influence insulin resistance and the incidence of diabetes. Therefore, visceral fat has been found to be an independent risk factor of metabolic syndrome, diabetes, and cardiovascular diseases [2]. Thus, recent studies have shown that the visceral fat might have a role in the pathogenesis of cardiovascular disease. However, most studies on visceral fat focus on the abdominal area only. In fact, studies on the association of visceral fat in other regions of the body with diabetes are insufficient.

Epicardial fat (EF) is defined as the adipose tissue enclosed in the visceral pericardium including the fat surrounding coronary arteries [3]. EF originates from brown fat and has the same origin as visceral fat, which shares much of the pathophysiological properties of visceral fat [4]. Also EF secretes pro-inflammatory and anti-inflammatory cytokines and chemokines that directly contacts the surroundings of the coronary artery [3]. Although EF accounts for only 1% of total body fat, it has been suggested to influence the development of coronary atherosclerosis due to its endocrine and paracrine activities. Therefore, interest in EF is on the rise. Visceral fat, especially EF has been shown to have close correlation with insulin resistance [5]. For that reason, EF could be a major factor that influences metabolic diseases such as metabolic syndrome and cardiovascular diseases such as coronary artery disease. Several studies have reported the correlation between EF and insulin resistance [6-12]. Based on these study results, the hypothesis that EF may contribute to unfavorable metabolic risk profiles has been suggested [11].

Low dose chest computed tomography (LDCT) is a useful tool for lung cancer screening in health promotion centers. LDCT is frequently used these days due to the advantage that it does not require 6 hour fasting and the radiation exposure from LDCT is much less than the contrast-enhanced computed tomography (CT). EF thickness area and volume can be accurately measured by LDCT [4]. Among them, EF thickness can be most easily measured, has little difference in repeated measurement and EF thickness shows close correlation with the total EF volume [13]. Recently, many researchers have been interested in EF thickness and many studies were done. Although EF is distributed asymmetrically between the coronary artery and ventricle or between the ventricles, there were no differences by the measurement methods (ultrasonography or 3-dimensional heart computed tomography) in the thickness of EF in previous studies [14-16]. EF thickness can be measured at the level of the three main coronary arteries (right, left anterior descending and left circumflex arteries) or at the right ventricular free wall [4]. EF thickness, especially measured around the left main coronary artery-fat (LMCA-fat) has been used in many studies since it can be most simply measured using just few image slices. In this study, we investigate the association of LDCT-measured LMCA-fat thickness with the prevalence of type 2 diabetes in Korean men.

## Method

### Study population

The present study was a retrospective cross-sectional study to examine the association between EF and prevalence of diabetes in Korean men participating in a medical health check-up program at the health promotion center of Ewha Womans University School of Medicine Seoul, Korea. The subjects of this study were 1,101 adult males aged 20 or older who received LDCT scans for lung cancer screening between January 2011 and June 2012. Diabetes was defined as fasting blood glucose of more than 126 mg/dL, or HbA1c value of more than 6.5%, or current use of oral hypoglycemic agents or insulin [17]. Among the 1,101 participants, 53 men who had history of malignancy or cardiovascular disease and whose presence of diabetes could not be determined were excluded from the study. Accordingly, 1,048 participants were included in the final analysis. This study was approved by the Institutional Review Board of Ewha Woman's University Mokdong Hospital (IRB file number: EUMC 2014-05-022-001). Informed consent was obtained from all participants.

### Study method

Study data included medical history physical examination, information provided by questionnaires, anthropometric measurements and laboratory measurements. Medical history was assessed by the examining physicians. All the participants were asked about health-related behaviors including alcohol consumption and smoking. Questions about alcohol consumption included frequency of alcohol intake per week and usual amount of alcohol intake per day. Participants were considered as current smokers when reported to have smoked within the last six months, and nonsmokers when reported to have not smoked for the last six months or who have never smoked [18]. Their heights were measured down to the 0.1 cm unit with the subjects wearing a light gown for health examination, with their heels attached to the ground, and their back straightened. Their weights were measured wearing a light gown of less than 0.1 kg, after an overnight 8 hour fasting and after urination. Body mass index (BMI) was defined as their weight (kg) divided by the square of their height (m). Waist circumference (WC) was measured down to the 0.1 cm unit at the midpoint between the lower rib margin and the iliac crest. After resting for at least 5 minutes, blood pressures were measured using a standardized mercury sphygmomanometer at the right brachial artery while sitting on a chair. Systolic blood pressure (SBP) and diastolic blood pressure (DBP) were recorded as the first and fifth Korotkoff phases, respectively. The definition of hypertension followed those of NCEP ATPIII, JNC 8 (The Eighth Report of the Joint National Committee on Prevention, Detection, Evaluation, and Treatment of High Blood Pressure). According to the JNC-8 guidelines, hypertension was defined as cases of a systolic blood pressure of 140 mmHg or higher or a diastolic blood pressure of 90 mmHg or higher which were measured during the health examination or taking antihypertensive agent after being diagnosed with hypertension [19]. If the blood pressure was abnormal at first measurement, they were measured once more after resting for 10 minutes and the latter blood pressure was used as study data.

### Laboratory measure

For the blood tests peripheral venous samples were collected after fasting for at least 8 hours. Total cholesterol, triglyceride (TG), low density lipoprotein (LDL) cholesterol, high density lipoprotein (HDL) cholesterol, aspartate aminotransferase (AST), alanine aminotransferase (ALT), gamma galutamyltransferase (GGT), creatinine, fasting blood glucose, and hemoglobin A1c (HbA1c) were measured. These levels were measured using Bayer Reagent Packs (Bayer HealthCare, Tarrytown, NY, USA) with an automated chemistry analyzer (ADVIA 1650 Autoanalyzer Bayer Diagnostics Leverkusen, Germany). The clinical laboratory has been accredited and participates annually in inspections and surveys done by the Korean Association of Quality Assurance for Clinical Laboratories.

### LMCA-fat thickness measurement

In this study LDCT scan was taken using a 128 multi-detector CT scanner (SOMATOM Definition Flash 128, Siemens Medical Solution, Forchheim, Germany). Each scan was obtained during a single breath-hold. The thickness of each slice was 2 mm, average scan duration was 4–6 seconds and the tube voltage was low dose (120 kVp, 20 eff.mAs). EF was defined as adipose tissue between the surface of the myocardium and the visceral layer of the pericardium. The LMCA-fat thickness was measured at the longest vertical length of EF around the left main coronary artery at the origin and was measured by the same doctor. Since a single slice image is 2 mm in thickness, the longest length of LMCA-fat thickness was measured by looking at approximately 15 slices around the LMCA. LMCA-fat thickness was measured manually by one doctor and fat pixels were identified using threshold attenuation values of −30 to −200 HU [20].

### Statistical analysis

Data in the text and tables were expressed as mean ± standard deviation (SD) or medians (interquartile range) for continuous variables and percentages of the number for categorical variables. Participants were divided into quartiles on the basis of LMCA-fat thickness as follows: quartile 1 (Q1) LMCA-fat thickness < 12.3 mm; quartile 2 (Q2) LMCA-fat thickness 12.3 to 15.4 mm quartile 3 (Q3) LMCA-fat thickness 15.4 to 18.7 mm; and quartile 4 (Q4) LMCA-fat thickness ≥ 18.7 mm. One-way ANOVA and χ^2^-test were performed to analyze the statistical differences among the characteristics of the participants according to the quartile groups of LMCA-fat thickness. To analyze the correlation between LMCA-fat thickness and the related variables, Pearson correlation analysis was performed. Linear-by-linear association was used to analyze the differences in the prevalence of diabetes by LMCA-fat thickness quartiles with p for trend. Compared to the lowest quartile of LMCA-fat thickness, the Odds ratios (ORs) of the highest 3 quartiles of LMCA-fat thickness were calculated using logistic regression. Also, logistic regression was used to analyze the independent risk of LMCA-fat thickness on diabetes after adjusting the clinical variables related to diabetes such as age, BMI, SBP, DBP, AST, ALT, TG, HDL cholesterol, and LDL cholesterol. PASW Statistics version 18.0 for Windows (SPSS Inc., Chicago, IL, USA) was used for statistical analyses. *P* values of less than 0.05 were considered to be statistically significant in every analysis.

## Results

The demographic and biochemical characteristics of the participants according to type 2 diabetes are presented in Table 1. Among the total of 1,048 subjects 13.5% (141 participants) were found to have diabetes. The mean age of study participants was 41.3 ± 6.3 years, 20.7% (217 participants) were current smokers and 17.8% (187 participants) were patients with hypertension. The mean BMI and WC of study participants were 24.9 ± 7.3 kg/m^2^ and 88.4 ± 5.6 cm, respectively. Participants with diabetes were older, more obese, smokers, had higher blood pressure and unhealthy laboratory findings. In these two groups, there were no significant differences in total cholesterol and LDL cholesterol. LMCA fat thickness in the diabetes group (17.6 ± 6.7 mm) was significantly higher than in the non-diabetes group (14.4 ± 5.9 mm) (p < 0.001).

**Table 1:** **Baseline characteristics of the study population (n = 1,048)**

**Variable**	**Overall (n = 1,048)**	**Diabetes (n = 141)**	**Non-diabetes (n = 907)**	p value*
Age	41.3 ± 6.3	44.1 ± 7.1	40.25 ± 6.9	<0.001
Smoker (%)	217 (20.7)	39 (27.7)	178 (19.6)	<0.001
Hypertension (%)	187 (17.8)	37 (26.2)	150 (16.5)	<0.001
BMI (kg/m^2^)	24.9 ± 7.3	26.1 ± 5.7	23.7 ± 2.8	<0.001
WC (cm)	88..4 ± 5.6	91.2 ± 6.1	86.5 ± 7.5	<0.001
SBP (mmHg)	125.3 ± 12.4	126.1 ± 10.5	124.8 ± 11.4	0.024
DBP (mmHg)	75.4 ± 9.6	77.2 ± 10.1	75.1 ± 10.0	<0.001
AST (IU/L)	24.7 ± 7.9	26.2 ± 8.5	24.2 ± 6.5	0.007
ALT (IU/L)	26.2 ± 8.1	27.5 ± 7.1	25.6 ± 9.4	0.006
GGT(IU/L)	41.5 ± 8.5	43.6 ± 8.2	40.2 ± 10.1	<0.001
Total cholesterol (mg/dL)	189.7 ± 29.5	190.1 ± 28.7	189.6 ± 30.8	0.751
Triglyceride (mg/dL)	127.6 ± 24.9	131.7 ± 25.1	127.1 ± 19.9	<0.001
HDL-C(mg/dL)	48.4 ± 10.4	48.0 ± 9.8	49.6 ± 11.1	0.021
LDL-C (mg/dL)	119.3 ± 30.3	120.4 ± 29.2	119.1 ± 33.5	0.252
Fasting glucose (md/dL)	97.5 ± 20.1	103.8 ± 21.5	96.1 ± 25.4	<0.001
HbA1c (%)	5.9 ± 1.1	6.3 ± 1.2	5.7 ± 0.9	<0.001
LMCA fat (mm)	14.9 ± 5.3	17.6 ± 6.7	14.4 ± 5.9	< 0.001

*P value was calculated by ANOVA or chi-square test.

Abbreviation: LMCA. left main coronary artery fat distance measured by low dose chest CT, DM, diabetes mellitus.

Table 2 shows clinical characteristics according to the quartile groups of LMCA-fat thickness. As expected age, hypertension, BMI, WC, and blood pressure of the participants tended to increase according to the quartile groups. The prevalence of diabetes also tended to increase according to the quartile groups of LMCA-fat thickness level (p < 0.001). All clinical variables showed statistically significant differences between the four groups except for recent smoking status. The higher LMCA quartiles had less favorable metabolic profiles. In the laboratory findings, there were significant linear trends across the quartiles of LMCA except for total cholesterol and LDL-cholesterol. Age, prevalence of hypertension, BMI, WC, blood pressure, AST, ALT, GGT, Triglyceride, fasting glucose, HDL cholesterol and HbA1c tended to increase as the LMCA fat thickness level increased. Differences in the prevalence of diabetes were examined through p for trend analysis according to the LMCA-fat thickness quartiles and are presented at the bottom of Table 2. A total of 141 men were identified to have diabetes by fasting glucose, HbA1c and medical history among the 1,048 participants. When the LMCA fat thickness was divided into four quartiles, the prevalence of diabetes was 6.9% (18 participants) in quartile 1, 9.5% (25 participants) in quartile 2, 16.4% (43 participants) in quartile 3 and 21.3% (55 participants) in quartile 4. In other words, there was a significant linear trend in the prevalence of diabetes across the quartiles of LMCA-fat thickness level. As expected, the prevalence of diabetes tended to increase depending on the LMCA fat thickness (p for trend < 0.001).

**Table 2:** **Clinical characteristics of the study population and LMCA fat quartile**

**Variable**	**LMCA fat quartile**				p value*
	**Q1**	**Q2**	**Q3**	**Q4**	
Age (years)	36.9 ± 9.5	40.5 ± 9.2	42.6 ± 9.8	43.4 ± 10.3	<0.001
Smoker (%)	51 (19.7)	47 (18.1)	39 (15.6)	80 (30.6)	0.091
Hypertension (%)	34 (12.9)	41 (15.5)	53 (20.4)	59 (22.4)	0.001
BMI (kg/m^2^)	23.4 ± 3.9	24.7 ± 4.2	25.3 ± 3.8	26.0 ± 3.1	0.014
WC (cm)	87.4 ± 5.9	88.1 ± 6.7	88.9 ± 6.5	90.6 ± 7.1	<0.001
SBP (mmHg)	122.6 ± 12.1	125.1 ± 12.2	125.6 ± 11.6	127.7 ± 12.2	<0.001
DBP (mmHg)	74.4 ± 9.3	75.6 ± 10.2	76.9 ± 10.0	78.3 ± 9.5	<0.001
AST (IU/L)	22.3 ± 7.3	23.5 ± 8.1	25.2 ± 6.9	26.4 ± 7.9	0.004
ALT (IU/L)	22.4 ± 8.2	25.5 ± 7.5	27.1 ± 9.0	27.3 ± 8.3	0.010
GGT(IU/L)	31.6 ± 9.3	36.3 ± 8.6	43.5 ± 10.1	45.2 ± 11.3	<0.001
Total cholesterol (mg/dL)	188.7 ± 33.8	191.3 ± 31.7	187.6 ± 34.8	190.4 ± 35.9	0.901
Triglyceride (mg/dL)	106 ± 21.9	116 ± 23.1	130 ± 19.4	128 ± 21.3	<0.001
HDL-C(mg/dL)	51.5 ± 11.4	49.8 ± 10.4	48.6 ± 11.5	49.5 ± 11.0	0.017
LDL-C (mg/dL)	121.3 ± 32.3	123.4 ± 29.1	119.1 ± 33.1	119.3 ± 33.6	0.214
Fasting glucose (md/dL)	90.2 ± 21.8	93.8 ± 20.9	96.2 ± 22.1	97.5 ± 18.6	<0.001
HbA1c (%)	5.8 ± 1.0	5.9 ± 0.9	6.0 ± 0.8	6.1 ± 0.8	0.002
Diabetes (%)	18 (6.9)	25 (9.5)	43 (16.4%)	55 (21.3%)	<0.001

*P value was calculated by ANOVA or chi-square test.

Data are presented as mean ± standard deviation.

LMCA fat: left main coronary artery fat distance measured by low dose chest CT; BMI: Body mass index; WC: Waist circumference; SBP: systolic blood pressure; DBP: diastolic blood pressure; AST: asparatate transaminase; ALT: alanine aminotransferase; GGT: gamma galutamyl transferase; HDL-C: high density lipoprotein cholesterol; LDL-C: low density lipoprotein cholesterol; HBA1c: hemoglobin A1c; DM: diabetes mellitus.

To analyze the correlation between LMCA fat thickness and the related variables a Pearson correlation analysis was performed (Table 3). LMCA fat thickness correlated with BMI (r = 0.169, p = 0.004) and WC (r = 0.172, p < 0.001). Fasting glucose (r = 0.105, p = 0.037) and HbA1c (r = 0.176, p < 0.001) showed significant positive correlation with the LMCA fat thickness. Age, blood pressure and most laboratory findings except for fasting glucose and HbA1c did not have significant correlations with LMCA fat thickness.

**Table 3:** **Pearson correlation between LMCA fat and clinical parameter**

**Variable**	**LMCA fat**	
	r*	**p value†**
Age	−0.005	0.892
BMI	0.169	0.004
WC	0.172	<0.001
SBP	0.064	0.145
DBP	0.030	0.413
AST	0.008	0.856
ALT	0.011	0.794
GGT	0.023	0.453
Total cholesterol	−0.009	0.723
Triglyceride	0.029	0.713
HDL-C	−0.024	0.647
LDL-C	−0.009	0.759
Fasting glucose	0.106	0.037
HbA1c	0.176	<0.001

*r means pearson correlation coefficient.

† P value was calculated by pearson correlation.

LMCA fat: left main coronary artery fat distance measured by low dose chest CT; BMI: body mass index; WC: waist circumference; SBP: systolic blood pressure; DBP: diastolic blood pressure; AST: asparatate transaminase; ALT: alanine aminotransferase; GGT: gamma galutamyl transferase; HDL-C: high density lipoprotein cholesterol: LDL-C: low density lipoprotein; HBA1c: hemoglobin A1c.

The unadjusted OR of diabetes were 1.43 (95% CI = 0.76-2.69) in quartile 2 2.66 (95% CI = 1.49-4.75) in quartile 3 and 3.69 (95% CI = 2.10-6.47) in quartile 4 compared to quartile 1 (Table 4). Even after adjustment for age, BMI, WC, TG, systolic BP, GGT, ALT, smoking status and alcohol intake, the prevalence of diabetic in quartile 4 still showed statistically significant higher levels compared with quartile 1. But after adjustment for the above mentioned factors, the OR of diabetes were 1.63 (95% CI = 0.57-4.65) in quartile 2 and 1.51 (95% CI = 0.52-4.39) in quartile 3, respectively. However, we saw that increased LMCA fat thickness was significantly associated with higher odds of type 2 diabetes (p for trend = 0.022).

**Table 4:** Odds ratio of diabetes according to LMCA fat quartile*

	**Odds ratio ( 95% Confidence Interval)**	
	**Unadjusted**	**Multivariate-adjusted model†**
LMCA		
Quartile 1	1.00 (reference)	1.00 (reference)
Quartile 2	1.43 (0.76-2.69)	1.63 (0.57-4.65)
Quartile 3	2.66 (1.49-4.75)	1.51 (0.52-4.39)
Quartile 4	3.69 (2.10-6.47)	3.26 (1.17-9.12)
P for linear trend	<0.001	0.022
Age		1.05 (1.02-1.09)
BMI		1.08 (0.96-1.22)
WC		1.07 (1.01-1.08)
Triglyceride		1.00 (1.00-1.01)
Systolic BP		1.02 (1.00-1.05)
GGT		1.00 (1.00-1.01)
ALT		1.00 (0.99-1.01)
Smoking status		1.93 (0.82-4.54)
Alcohol intake		1.37 (0.52-3.62)

*Data are given as odds ratio (95% confidence intervals) by logistic regression analysis.

† Multivaiate data are adjusted by age, BMI, Triglyceride, systolic BP, GGT, ALT, smoking status and alcohol intake.

LMCA fat: left main coronary artery fat distance measured by low dose chest CT; 95% CI:95% confidence intervals; BMI: body mass index; WC: waist circumference; BP: blood pressure; GGT: gamma galutamyl transferase; ALT: alanine aminotransferase.

## Discussion

### Main results

The result of this study showed significant association between LMCA fat thickness and the prevalence of diabetes in Korean men. In this study the prevalence of diabetes was increased in a dose-dependent manner by increasing quartiles of LMCA-fat thickness (p for trend < 0.001). In other words, the prevalence of diabetes increased as the EF thickness increased. According to the quartiles of LMCA fat thickness, the prevalence of diabetes in quartile 2, quartile 3, and quartile 4 were 1.43 times, 2.66 times, and 3.69 times higher respectively, than that of quartile 1 which has the lowest EF thickness. This indicates that the increase of EF thickness is associated with the increase of diabetes prevalence. This association persisted after adjusting for age, BMI, WC, TG, SBP, GGT, ALT, smoking status and alcohol intake in quartile 4 which has the highest EF thickness.

### Potential mechanisms

The relationship between EF and diabetes can be explained in a few ways. First EF is the visceral fat surrounding the heart and is closely correlated with abdominal fat [21]. The relationship can be explained with by the developmental process of the fetus [5]. Both EF and abdominal fat seem to originate from brown fat which has less anti-lipolytic effect of insulin. Thus, EF is expected to have the same biochemical characteristics as abdominal fat and play a key role in the pathogenesis of diabetes and insulin resistance [7]. EF thickness was shown to be significantly and independently associated with left ventricular diastolic dysfunction and inflammation in patients undergoing peritoneal dialysis and these associations might act synergistically in the pathogenesis of unfavorable metabolic profiles [22]. Moreover, it is known that EF secretes inflammation-related materials such as tumor necrosis factor-alpha (TNF-α), interleukine-6 (IL-6), free fatty acids (FFA), plasminogen activator inhibitor-1 (PAI-1) and adiponectin into blood vessels, especially the coronary arteries, thereby influencing diabetes, obesity, and dyslipidemia [23]. In fact, EF surrounding the coronary arteries might secrete adipokines such as adiponectin from ‘outside-to-inside’ into the intima-media layer of blood vessels, thereby contributing to inflammation, atherosclerosis and insulin resistance. Considering the circulation system of our body, it is possible to explain that inflammatory cytokines will be delivered to the whole body because the heart supplies blood to the whole body. Increased plasma concentrations of TNF-α, IL-6, FFA, and PAI-1 and decreased concentrations of adiponectin may lead to accelerated atherosclerosis, plaque instability, and arterial thrombosis. Interestingly, a recent study has shown that FABP4 (Fatty acid-binding proteins 4) expressed in adipocytes or macrophages via classical secretion of numerous inflammation–related materials act as an adipokine for the development of insulin resistance [24]. In addition, it has been shown that higher retinol-binding protein 4 (RBP4) and lower glucose transporter 4 (GLUT4) might contribute to an unfavorable metabolic condition including insulin resistance [25]. RBP4 as an adipokine has been shown to induce insulin resistance and GLUT4 to mediate insulin-stimulated glucose uptake in EF. These inflammatory substances of EF are thought to have significant inflammatory and proatherogenic effects. Up to date, studies are not able to reveal the direct effects of EF on cardiovascular diseases and diabetes.

### Comparision with other studies

Several studies have reported of the association between the EF surrounding coronary arteries and heart and the development of metabolic syndrome and coronary atherosclerosis. Petra M. Gorter et al. reported that epicardial fat and pericoronary fat are associated with the existence of obesity and metabolic syndrome in patients with coronary artery disease [13]. Iacobelli G et al. revealed that EF measured by transthoracicechocardiography could be a potential risk factor for explaining fasting blood glucose disorder [21]. Although Iacobelli G et al. showed limited results on the correlation between EF and fasting blood glucose [21], our study is meaningful in that the association between EF and the prevalence of diabetes was examined directly. When compared with other studies our study results show a relatively low correlation between BMI and EF thickness (r = 0.169, p value = 0.004). This may be due to the fact that the participants were from health check-ups, so they were much interested in their health and also to the fact that they were relatively young. The reason that other studies have shown a more higher association with BMI may be due to the fact that in most studies researchers investigated EF of patients with coronary heart disease [8,9,13,17,26] or gestational diabetes [27]. In contrast, some studies reported that EF was significantly higher in patients with coronary artery disease or left ventricular diastolic dysfunction but BMI had no statistical significance. Despite this study being consisted of more young and healthy people, we showed significant association between EF thickness and the prevalence of diabetes in Korean men after adjustment for BMI and WC. This is meaningful in that the participants of our study were those without previous heart diseases. The important point is that high EF was a more important factor than high weight or high visceral fat in healthy Korean men even though the correlation coefficient r value was lower than other studies.

### Limitations of this study

Nevertheless some limitations need to be taken into account as well. First, the pathophysiological effect of EF on predisposition to diabetes was not investigated. In this cross-sectional study, we found the simple correlation between EF and the prevalence of diabetes, so we could not find the causal relationship between them. Second, the study participants were limited to a single institution. In addition, because we limited the participants to those who received LDCT scan for a medical health check-up, there can be a problem in applying this result to the general population. Third, this study confirmed the association between EF thickness and diabetes but a volumetric analysis of EF was not performed. Since volumetric analysis of EF is a time consuming work and requires skilled operators and a specific software program for semi-automated measurement of EF volume, it is difficult to be easily done. Besides, EF thickness shows close correlation with the measurement of the total EF volume [13]. In addition, it is important that we could measure EF thickness in a very simple method without the help of other software programs.

## Conclusion

Our findings indicate that increased EF thickness – not abdominal fat or total body fat - is independently associated with the prevalence of diabetes in Korean men. In addition, EF could be considered a marker for unfavorable metabolic profile.

## Abbreviations

AST: Aspartate aminotransferase
ALT: Alanine aminotransferase
BMI: Body mass index
CT: Computed tomography
DBP: Diastolic blood pressure
EF: Epicardial fat
GGT: Γ-glutamyltransferase
HbA1c: Hemoglobin a1c
HDL: High-density lipofpdlsprotein
LDCT: Low dose chest computed tomography
LDL: Low-density lipoprotein
LMCA-fat: Left main coronary artery fat
SBP: Systolic blood pressure
TG: Triglyceride
WC: Waist circumference
